# Netrin-1 Derived from the Ventricular Zone, but not the Floor Plate, Directs Hindbrain Commissural Axons to the Ventral Midline

**DOI:** 10.1038/s41598-017-12269-8

**Published:** 2017-09-20

**Authors:** Kenta Yamauchi, Maya Yamazaki, Manabu Abe, Kenji Sakimura, Heiko Lickert, Takahiko Kawasaki, Fujio Murakami, Tatsumi Hirata

**Affiliations:** 1 Division of Brain Function, National Institute of Genetics, Mishima, Shizuoka, 411-8540 Japan; 2 Laboratory of Neuroscience, Graduate School of Frontier Biosciences, Osaka University, Suita, Osaka, 565-0871 Japan; 3 Department of Cellular Neurobiology, Brain Research Institute, Niigata University, Niigata, 951-8585 Japan; 4 Institute of Stem Cell Research and Institute of Diabetes and Regeneration Research, Helmholtz Zentrum München, D-85764 Neuherberg, Germany; 5 Present Address: Department of Neurology, University of California, San Francisco, San Francisco, CA 94143 USA

## Abstract

Netrin-1 (Ntn1) emanating from the ventral midline has been thought to act as a long-range diffusible chemoattractant for commissural axons (CAs). However, CAs still grow towards the midline in the absence of the floor plate (FP), a glial structure occupying the midline. Here, using genetically loss-of-function approaches in mice, we show that Ntn1 derived from the ventricular zone (VZ), but not the FP, is crucial for CA guidance in the mouse hindbrain. During the period of CA growth, *Ntn1* is expressed in the ventral two-thirds of the VZ, in addition to the FP. Remarkably, deletion of *Ntn1* from the VZ and even from the dorsal VZ highly disrupts CA guidance to the midline, whereas the deletion from the FP has little impact on it. We also show that the severities of CA guidance defects found in the *Ntn1* conditional mutants were irrelevant to their FP long-range chemoattractive activities. Our results are incompatible with the prevailing view that Ntn1 is an FP-derived long-range diffusible chemoattractant for CAs, but suggest a novel mechanism that VZ-derived Ntn1 directs CAs to the ventral midline by its local actions.

## Introduction

In bilaterally symmetrical organisms, commissural axons (CAs) projecting across the midline convey information from one side of nervous system to the other to connect both sides. Commissural neurons exist at all axial levels of the CNS and exhibit diverse projection patterns^1,2^. Among them, ventrally decussating commissural projections in the midbrain, hindbrain and spinal cord develop through a ventral midline structure, the floor plate (FP)^1,2^. These projections have been believed to be established by a common axon guidance mechanism, chemoattraction by a long-range diffusible molecule, Netrin-1 (Ntn1), emanating from the FP^3^.

Chemoattraction of CAs by way of establishing a gradient of a diffusible molecule emanating from the FP was initially postulated by Ramón y Cajal over a century ago^4^. In accordance with this idea, FP explants have been shown to attract CAs at a distance *in vitro* by secreting diffusible factors that influence their outgrowth and orientation^5–10^. Two Ntn proteins, Ntn1 and Ntn2, were purified from chick brains based on the ability to mimic the outgrowth-promoting activity of the FP^11,12^. *Ntn1* is expressed in the FP, whereas *Ntn2* is expressed in the ventral two-thirds of the ventricular zone (VZ) of the neural tube^11^. In mice, which have *Ntn1* but not *Ntn2*, *Ntn1* expression appears to be a composite of *Ntn1* and *Ntn2* expression in the chick^13^. Critically, *Ntn1* deficient mice exhibit profound CA guidance defects^13–16^. An antibody against Ntn proteins reveal a dorsoventral gradient of Ntn proteins along the path of CAs^17^. Collectively, these findings led to a model that Ntn1 is an FP-derived long-range diffusible chemoattractant for CAs. However, contrary to this model, CAs in the spinal cord as well as the hindbrain still grow ventrally and reach the midline in the absence of the FP^18–20^, raising an alternative possibility that Ntn1 protein of extra-FP origin directs CAs to the ventral midline.

Here, we revisit the chemoattraction model by studying CA growth in the mouse hindbrain (medulla oblongata). We used a range of *Ntn1* conditional mutants to determine the physiologically relevant source of Ntn1. Our results reveal that *Ntn1* expression in the VZ, but not the FP, is crucial for CA guidance to the midline. Our results fail to support the prevailing view that Ntn1 is an FP-derived long-range diffusible chemoattractant for CAs, but suggest that local actions of Ntn1 from the VZ direct CAs to the ventral midline.

## Results

### *Ntn1* is expressed beyond the FP in the developing mouse hindbrain

We focused on the source of Ntn1 proteins for CAs to revisit the idea that Ntn1 is an FP-derived long-range diffusible chemoattractant. If this model is correct, FP-derived Ntn1 should be essential for the CA guidance.

**Figure 1:**
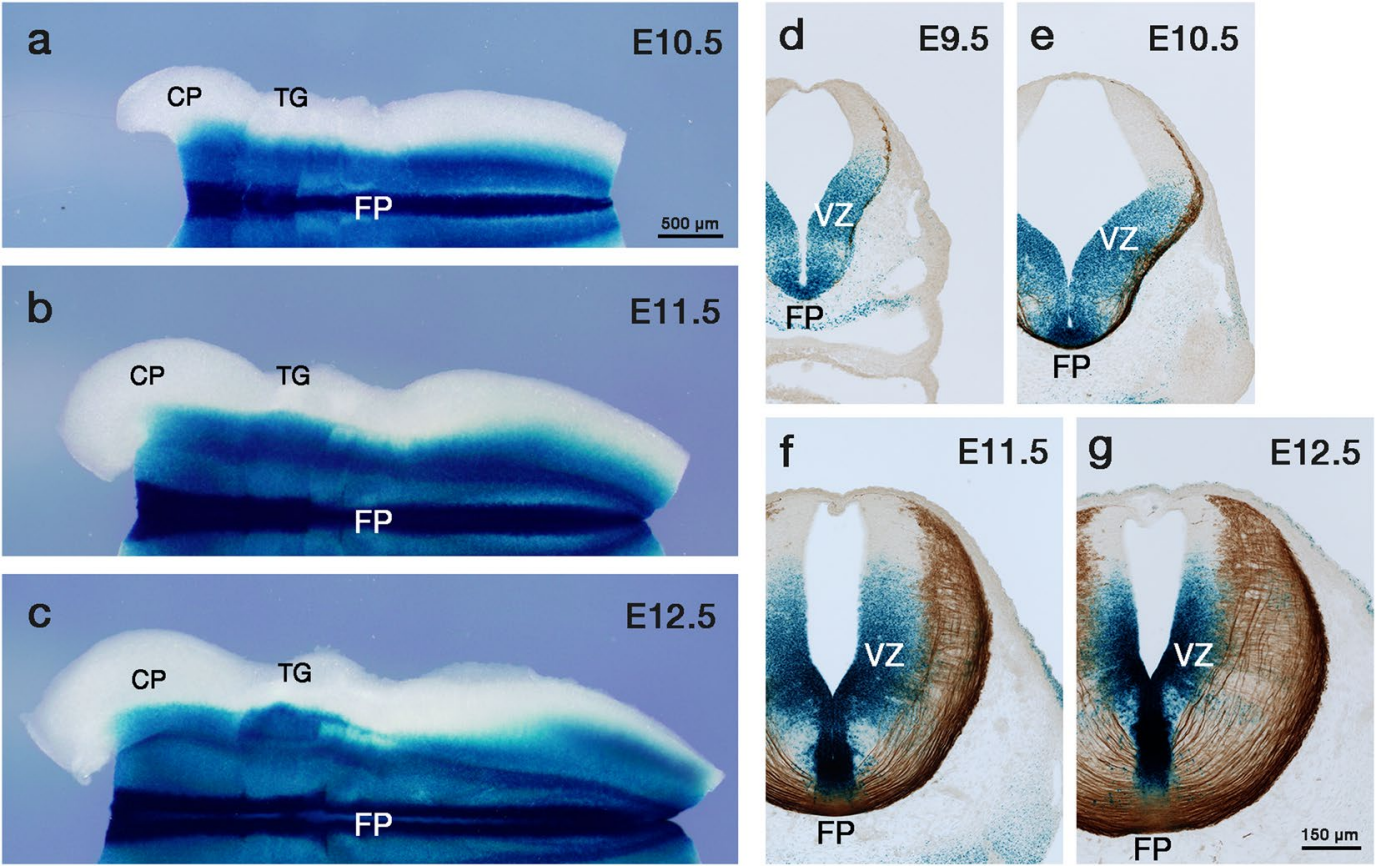
The spatiotemporal relationship between *Ntn1* expression and CA growth in the developing mouse hindbrain. (**a**–**c**) X-gal staining in whole-mount preparations of E10.5 (**a**), E11.5 (**b**) and E12.5 (**c**) *Ntn1* ^+/LacZ^ mouse hindbrains (E10.5, n = 12; E11.5, n = 8; E12.5, n = 8). The *Ntn1* ^+*/LacZ*^ mouse harbors a *ß*-*geo* gene trap vector^13^, allowing *Ntn1* expressions to depict by X-gal histochemistry. X-gal reaction products are found in the approximately ventral two-thirds of the hindbrain, in addition to the FP. Dorsal is upwards and rostral is towards the left. (**d**–**g**) X-gal histochemistry (blue) and Robo3 immunostaining (brown) in E9.5 (**d**), E10.5 (**e**) E11.5 (**f**) and E12.5 (**g**) *Ntn1* ^+*/LacZ*^ mouse hindbrain transverse sections at the rhombomere 7/8 level (E9.5, n = 4, E10.5, n = 4; E11.5, n = 3; E12.5, n = 3). At all these stages, X-gal products are detected in the FP and the ventral two-thirds of VZ along the entire or most part of the circumferential path of Robo3^+^ axons. Note that X-gal reaction products do not necessarily reflect Ntn1 protein localization. CP, cerebellar primodium; TG, trigeminal ganglia. The bar in (**a**) and (**g**) apply to (**a**–**c**) and (**d**–**g**), respectively.

Although *Ntn1* expression in the developing mouse hindbrain has been reported^14,21,22^, its expression during the period of CA growth towards the midline (embryonic day [E] 9.5 to E12.5) has yet to be fully described. We therefore examined *Ntn1* expression in the hindbrain using *Ntn1* ^*LacZ*^ mice, in which ß-galactosidase (ß-gal) expression represents endogenous *Ntn1* gene expression^13^. *Ntn1* was broadly expressed in the developing mouse hindbrain encompassing the FP; X-gal staining in whole-mount preparations showed prominent ß-gal activity in the approximately ventral two-thirds of the hindbrain (Fig. 1a–c). We then examined the spatiotemporal relationship between CA growth and *Ntn1* expression. For this, hindbrain sections from *Ntn1* ^+*/LacZ*^ mice were double labeled with X-gal and an antibody against Robo3, a marker for CAs projecting to the FP^23–26^. Robo3^+^ axons approaching the FP appeared at E9.5 (Fig. 1d) and the first cohort of the axons reached the FP by E10.5 (Fig. 1e). Robo3^+^ axons that had reached the FP markedly increased in number as development proceeds (E11.5–E12.5) (Fig. 1f,g). At all these stages, ß-gal activity was detected in the ventral two-thirds of the hindbrain; it was found in the VZ apical to the entire or most part of the circumferential path of Robo3^+^ axons, in addition to the FP (Fig. 1d–g). Intriguingly, ß-gal activity was largely absent from Robo3^+^ cells (Fig. 1d–g), suggesting that *Ntn1* transcription outside the FP mainly occurs in neuroepithelial cells. Thus, in the developing mouse hindbrain, Ntn1 protein synthesis occurs in the vicinity of the CA path, the VZ, in addition to the FP.

### Deletion of *Ntn1* from the VZ disrupts CA guidance

**Figure 2:**
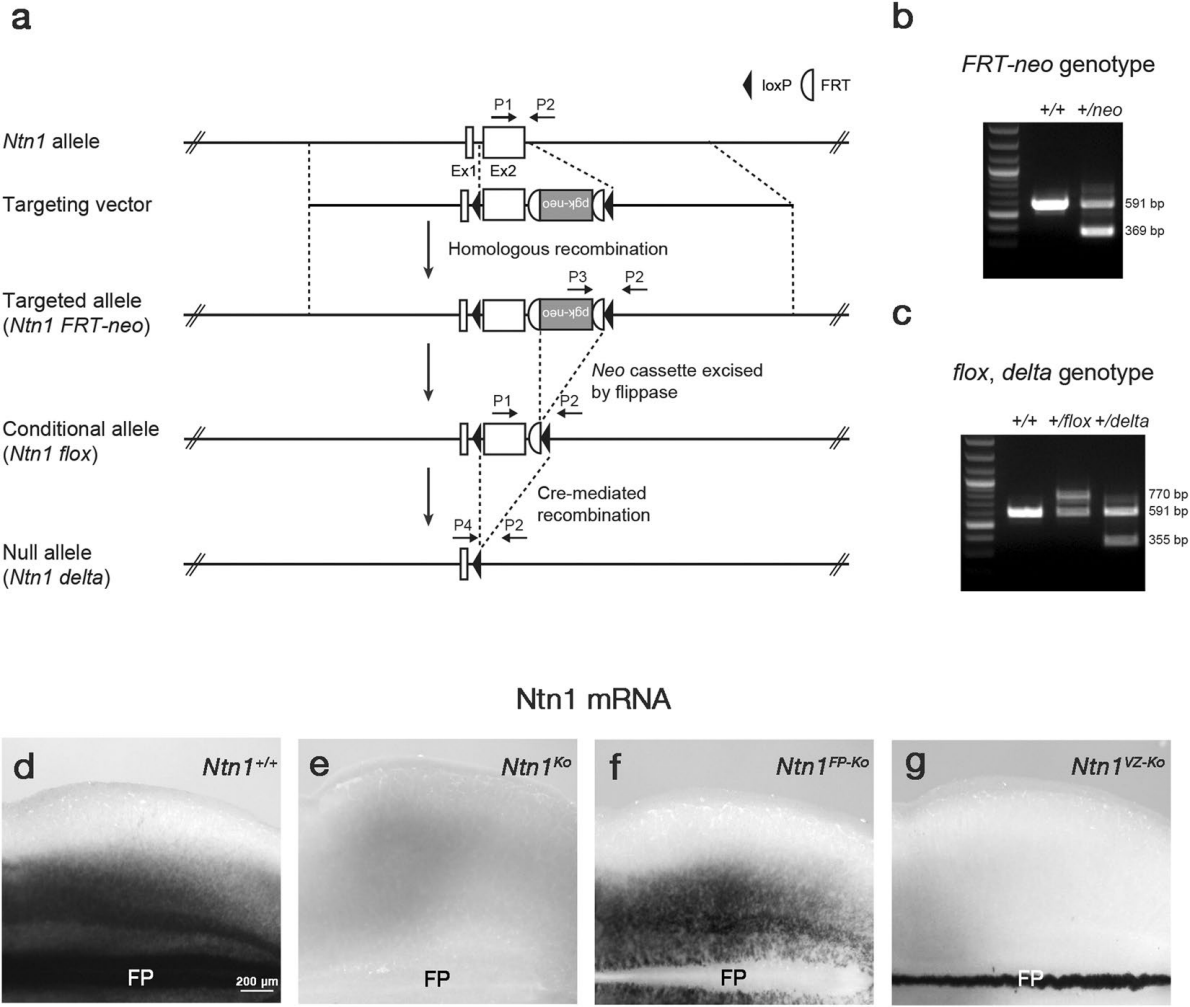
Generation of *Ntn1* ^*FP*-*Ko*^ and *Ntn1* ^*VZ*-*Ko*^ mice. (**a**) Targeting strategy for an *Ntn1* conditional allele. Schematic diagram of the *Ntn1* locus, targeting vector, targeted allele (*Ntn1* ^*FRT*-*neo*^), conditional allele (*Ntn1* ^*flox*^) and null allele (*Ntn1* ^*∆*^). *Ntn1* ^*flox*^ mice were generated by flanking the *Ntn1* exon 2 with *loxP* sites. (**b**,**c**) Multiplex PCR genotyping of the targeted allele (**b**) and the conditional and null allele (**c**) using primers P1 (*Ntn1 Frt Fw5*), P2 (*Ntn1 Frt Rv3*), P3 (*Pgk Pr2*) and P4 (*Ntn1 ∆ Fw2*). (**d**–**g**) *Ntn1* ISH in whole-mount preparations of E12.5 *Ntn1* ^+/+^ (**d**), *Ntn1* ^*Ko*^ (**e**), *Ntn1* ^*FP*-*Ko*^ (**f**) and *Ntn1* ^*VZ*-*Ko*^ (**g**) mouse hindbrains (*Ntn1* ^+/+^, n = 6; *Ntn1* ^*Ko*^, n = 5; *Ntn1* ^*FP*-*Ko*^, n = 5; *Ntn1* ^*VZ*-*Ko*^, n = 6). *Ntn1* is expressed in the FP and the ventral two-thirds of lateral neural tube in *Ntn1* ^+/+^ mice (**d**). *Ntn1* hybridization signals are not detected in *Ntn1* ^*Ko*^ mice (**e**). In *Ntn1* ^*FP*-*Ko*^ mice, *Ntn1* expression in the FP is deleted and that in the lateral domain is slightly reduced (**f**). The laterally expressed *Ntn1* is specifically deleted in *Ntn1* ^*VZ*-*ko*^ mice (**g**). Dorsal is upwards and rostral is towards the left. Hindbrains at the rhombomere 6–8 level are represented.

*Ntn1* expressions in the developing mouse hindbrain raise the question of which source is crucial for CA guidance to the midline. To address this question, we used conditional genetic approaches. A conditional allele of *Ntn1* (*Ntn1* ^*flox*^) was made by homologous recombination in ES cells (Fig. 2a–c). This allele was designed to delete the exon 2 from *Ntn1* gene. Cre-mediated recombination resulted in a null allele of *Ntn1* (*Ntn1* ^*∆*^) (Fig. 2e). The *Ntn1* ^*flox*^ mice were crossed with *Foxa2* ^*iCre*^ or *NestinCre* mice^27,28^ to generate *Ntn1* FP conditional mutant (*Foxa2* ^+*/iCre*^ *;Ntn1* ^*∆/flox*^ mice, denoted *Ntn1* ^*FP*-*Ko*^ mice hereafter) or VZ conditional mutant mice (*NestinCre;Ntn1* ^*flox/flox*^ mice, denoted *Ntn1* ^*VZ*-*Ko*^ mice hereafter), respectively. *In situ* hybridization (ISH) for *Ntn1* in E12.5 hindbrain whole-mount preparations confirmed the specific deletion of *Ntn1* from the FP or VZ in these mice (Fig. 2f,g). In the *Ntn1* ^*FP*-*Ko*^ mice, *Ntn1* expression in the lateral hindbrain was also slightly reduced (Fig. 2f). The recombination efficiencies in the *Foxa2* ^*iCre*^ and *NestinCre* mice were further assessed using *Z/EG* reporter mice^29^. Consistent with a previous study^28^, *Foxa2* ^*iCre*^ mice exhibited recombination in the FP by E9.5, a stage when CAs begin to project ventrally (Supplementary Fig. S1). In the *NestinCre* mice, partial recombination was detected at E10.5 and recombination throughout the entire VZ had been completed by E11.5 (Supplementary Fig. S2). Thus, we succeeded in generating tools that allow us to assess the roles of Ntn1 of FP and extra-FP origin.

**Figure 3:**
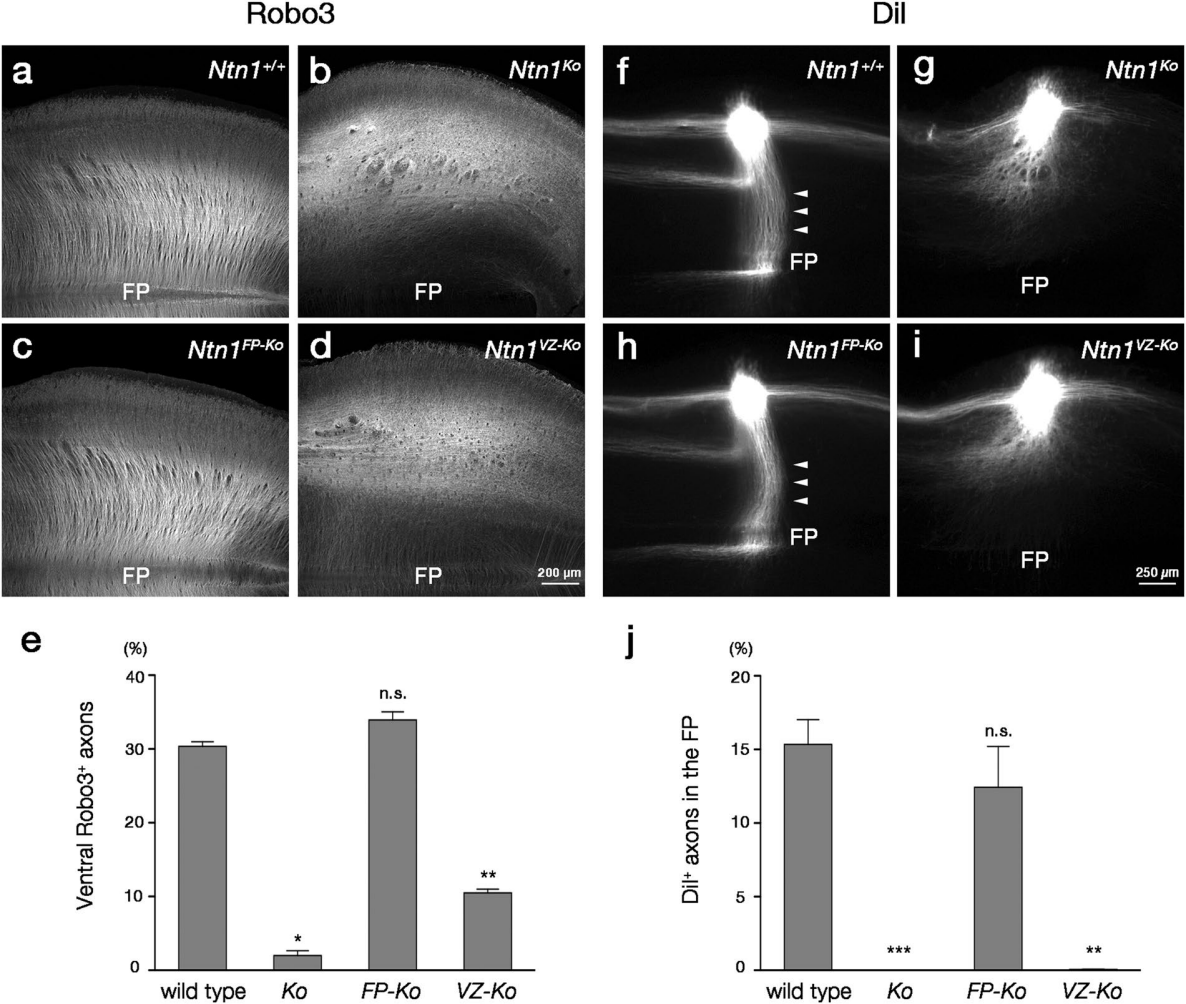
Aberrant CA growth caused by deletion of *Ntn1* from the VZ. (**a**–**d**) Robo3 immunostaining in whole-mount preparations of E12.5 *Ntn1* ^+/+^ (**a**), *Ntn1* ^*Ko*^ (**b**), *Ntn1* ^*FP*-*Ko*^ (**c**) and *Ntn1* ^*VZ*-*Ko*^ (**d**) mouse hindbrains (*Ntn1* ^+/+^, n = 7; *Ntn1* ^*Ko*^, n = 5; *Ntn1* ^*FP*-*Ko*^, n = 8; *Ntn1* ^*VZ*-*Ko*^; n = 7). Robo3^+^ axons grow ventrally and reach the FP in *Ntn1* ^+/+^ and *Ntn1* ^*FP*-*Ko*^ mice (**a**,**c**). Ventrally growing Robo3^+^ axons were highly disorganized and their number is markedly reduced in *Ntn1* ^*Ko*^ and *Ntn1* ^*VZ*-*Ko*^ mice (**b**,**d**). (**e**) Histograms representing the fluorescence intensity of Robo3^+^ axons within the ventral one-fourth of the hindbrain normalized to that in the preparation (*Ntn1* ^+/+^, n = 7; *Ntn1* ^*Ko*^, n = 5; *Ntn1* ^*FP*-*Ko*^, n = 8; *Ntn1* ^*VZ*-*Ko*^, n = 7; ^n.s^ *P* > 0.05, *P < 0.05, **P < 0.01; Kruskal-Wallis test followed by Steel *post hoc* test; *P*_*Ko*_ = 0.0126, *P*_*FP*-*Ko*_ = 0.159, *P*_*VZ*-*Ko*_ = 0.00493). Error bars indicate SEM. (**f–i**) DiI labeling of CAs in whole-mount preparations of E12.5 *Ntn1* ^+/+^ (**f**), *Ntn1* ^*ko*^ (**g**), *Ntn1* ^*FP*-*ko*^ (**h**) and *Ntn1* ^*VZ*-*ko*^ (**i**) mouse hindbrains (*Ntn1* ^+/+^, n = 6; *Ntn1* ^*Ko*^, n = 9; *Ntn1* ^*FP*-*Ko*^, n = 9; *Ntn1* ^*VZ*-*Ko*^, n = 8). DiI crystals are implanted into the dorsal hindbrain at the rhombomere 7/8 level. In *Ntn1* ^+/+^ mice, DiI-labeled CAs grow straight towards the FP (arrowheads) and crossed it (**f**). Longitudinally extending axons in the dorsal margin and ipsilaterally turning axons at the middle along the dorsoventral axis are also normally labeled (**f**). In *Ntn1* ^*Ko*^ mice, ventrally directed axons are foreshortened, spread rostrocaudally and fail to invade ventral hindbrain (**g**). *Ntn1* ^*FP*-*Ko*^ mice exhibit axon trajectories almost identical to those of *Ntn1* ^+/+^ mice (**h**, arrowheads), whereas *Ntn1* ^*VZ*-*Ko*^ mice exhibit CA guidance defects similar to those of *Ntn1* ^*Ko*^ mice (**i**). (**j**) Histograms representing the fluorescence intensity of DiI-labeled axons within the FP normalized to that at the implantation site (*Ntn1* ^+/+^, n = 6; *Ntn1* ^*Ko*^, n = 9; *Ntn1* ^*FP*-*Ko*^, n = 9; *Ntn1* ^*VZ*-*Ko*^, n = 8; ^n.s^ *P* > 0.05, **P < 0.01, ***P < 0.001; Kruskal-Wallis test followed by Steel *post hoc* test; *P*_*Ko*_ = 8.65 × 10^−4^, *P*_*FP*-*Ko*_ = 0.334, *P*_*VZ*-*Ko*_ = 0.00533). Error bars indicate SEM. Dorsal is upwards and rostral is towards the left. Hindbrains at the rhombomere 6–8 level are represented. The bar in (**d**) and (**i**) apply to (**a**–**d**) and (**f**–**i**), respectively.

We then stained E12.5 hindbrain whole-mount preparations with an anti-Robo3 antibody to visualize CA trajectories in these mutants (Fig. 3a–d). While Robo3^+^ axons grew ventrally and reached the midline, forming a consecutive stripe pattern in wild-type mice (Fig. 3a), in *Ntn1* null mice (*Ntn1* ^*∆/∆*^ mice, denoted *Ntn1* ^*Ko*^ mice hereafter), these axons were highly disrupted in the dorsal hindbrain and rarely directed ventrally (Fig. 3b,e). Surprisingly, deletion of *Ntn1* from the FP had little impact on the CA guidance; the ventrally directed growth and decussation were preserved in *Ntn1* ^*FP*-*Ko*^ mice (Fig. 3c, *P* = 0.159; Fig. 3e). In stark contrast, VZ-specific deletion caused striking abnormalities in CA growth; in *Ntn1* ^*VZ*-*Ko*^ mice, Robo3^+^ axons were highly disorganized in the dorsal hindbrain, resulting in a significant reduction in the number of the ventrally directed axons (Fig. 3d,e). These observations were further confirmed by labeling of CAs using a fluorescent lipophilic dye, DiI, implanted into the dorsal hindbrain (Fig. 3f–j). As was the case for Robo3 immunostaining, *Ntn1* ^*FP*-*Ko*^ mice exhibited axon trajectories almost identical to those of wild-type mice; DiI-labeled CAs grew straight towards the FP and crossed it (Fig. 3f,h, arrowheads). Although axons deflecting from the CA bundle were faintly visible near the FP in *Ntn1* ^*FP*-*Ko*^ mice (Supplementary Fig. S3), the proportion of DiI-labeled axons that had reached the FP was not significantly different between wild-type and *Ntn1* ^*FP*-*Ko*^ mice (*P* = 0.334; Fig. 3i). In contrast, *Ntn1* ^*VZ*-*Ko*^ mice exhibited CA misguidance similar to that found in *Ntn1* ^*Ko*^ mice; DiI-labeled CAs were foreshortened, spread rostrocaudally and most of them failed to invade the ventral hindbrain (Fig. 3g,i). Further analysis of higher magnification images revealed that, in both *Ntn1* ^*Ko*^ and *Ntn1* ^*VZ*-*Ko*^ mice, DiI-labeled axons were rostrally deflected in the ventral hindbrain (Supplementary Fig. S4). Together, these results indicate that *Ntn1* expression in the VZ, but not the FP, is crucial for the CA guidance.

### Ntn1 from the dorsal VZ is required for CA guidance

**Figure 4:**
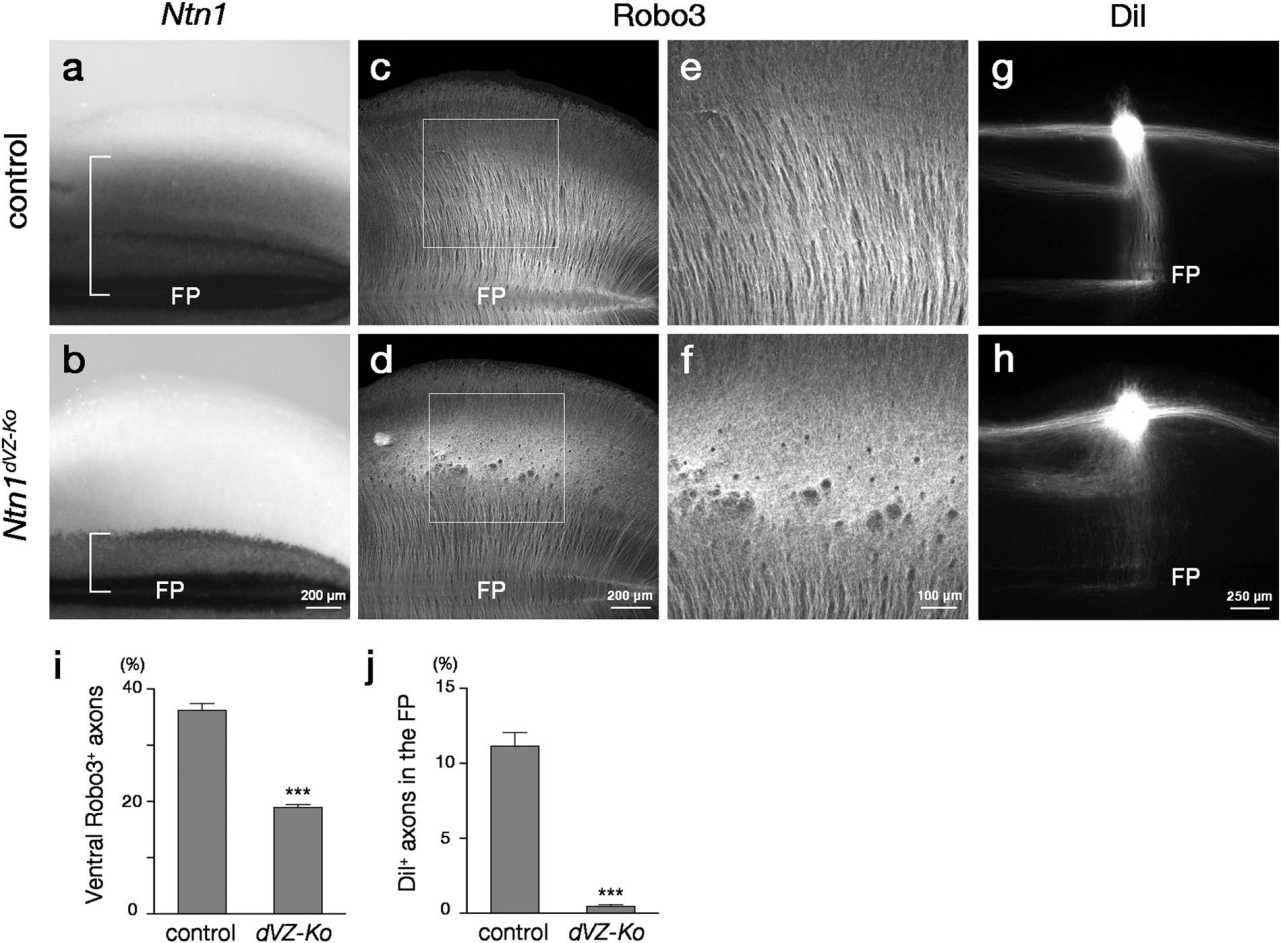
CA guidance defects caused by deletion of *Ntn1* from the dorsal VZ. (**a**,**b**) *Ntn1* ISH in whole-mount preparations of E12.5 control (*Pax3* ^+*/Cre*^ *;Ntn1* ^+*/flox*^) (**a**) and *Ntn1* ^*dVZ*-*Ko*^ (**b**) mouse hindbrains (control, n = 3; *Ntn1* ^*dVZ*-*Ko*^, n = 7). Dorsal expression of *Ntn1* is deleted in *Ntn1* ^*dVZ*-*Ko*^ mouse hindbrains. Brackets enclose *Ntn1* expression along the dorsoventral axis in each genotype. (**c**,**d**) Robo3 immunostaining in whole-mount preparations of E12.5 control (**c**) and *Ntn1* ^*dVZ*-*Ko*^ (**d**) mouse hindbrains (control, n = 10; *Ntn1* ^*dVZ*-*Ko*^, n = 9). (**e**) and (**f**) are enlarged views of the dorsal hindbrain regions in (**c**) and (**d**), respectively. In *Ntn1* ^*dVZ*-*Ko*^ mice, Robo3^+^ axons are disorganized in the dorsal hindbrain, reducing the number of ventrally growing Robo3^+^ axons. (**g**,**h**) DiI labeling of CAs in whole-mount preparations of E12.5 control (**g**) and *Ntn1* ^*dVZ*-*Ko*^ (**h**) mouse hindbrains (control, n = 9; *Ntn1* ^*dVZ*-*Ko*^, n = 7). DiI-labeled CAs originated from the dorsal hindbrain are defasciculated and often fail to reach the midline in *Ntn1* ^*dVZ*-*Ko*^ mice (**h**). (**i**,**j**) Histograms representing the fluorescence intensity of Robo3^+^ axons within the ventral one-fourth of the hindbrain normalized to that in the preparation (control, n = 10; *Ntn1* ^*dVZ*-*Ko*^, n = 9; ***P < 0.001, Mann–Whitney U-test; *P* = 2.17 × 10^−5^) (**i**) and the fluorescence intensity of DiI-labeled axons within the FP normalized to that at the implantation site (control, n = 9; *Ntn1* ^*dVZ*-*Ko*^, n = 7; ***P < 0.001, Mann–Whitney U-test; *P* = 1.75 × 10^−4^) (**j**), respectively. Error bars indicate SEM. Dorsal is upwards and rostral is towards the left. Hindbrains at the rhombomere 6–8 level are represented. The bar in (**b**), (**d**), (**f**) and (**h**) apply to (**a**,**b**), (**c**,**d**), (**e**,**f**) and (**g**,**h**), respectively.

*Ntn1* expression extended to the dorsal VZ where most commissural neurons differentiate (Fig. 1d–g). We wondered whether the Ntn1 derived from the region of commissural neurons generation is involved in the CA guidance. To explore this possibility, *Ntn1* ^*flox*^ mice were crossed with *Pax3* ^*Cre*^ mice^30^ to generate *Ntn1* dorsal VZ conditional mutant mice (*Pax3* ^+*/Cre*^; *Ntn1* ^*∆/flox*^ mice, denoted *Ntn1* ^*dVZ*-*Ko*^ mice hereafter), as *Pax3* is specifically expressed in the dorsal neural tube of the hindbrain^31^. ISH for *Ntn1* in E12.5 hindbrain whole-mount preparations confirmed deletion of *Ntn1* from the dorsal neural tube in the mutants (Fig. 4a,b). As reported previously^30^, Cre-mediated recombination in the dorsal neural tube of the *Pax3* ^*Cre*^ mice occurred by E9.5, a stage when CAs start to extend ventrally (Supplementary Fig. S4). CA trajectories in *Ntn1* ^*dVZ*-*Ko*^ mice were examined with Robo3 immunostaining and DiI labeling (Fig. 4c–j). Remarkably, ventrally directed Robo3^+^ axons were disorganized and their number was reduced in *Ntn1* ^*dVZ*-*Ko*^ mice (Fig. 4c–f,i). Furthermore, DiI-labeled CAs were defasciculated and often failed to reach the FP (Fig. 4g,h,j). Thus, *Ntn1* expression in the dorsal VZ is required for the CA guidance, highlighting local actions of Ntn1. CA guidance defects caused by deletion of *Ntn1* from the dorsal VZ were less severe than those caused by deletion of *Ntn1* from the entire VZ (compare Figs 3d,e,i,j with 4d,h,i,j), suggesting that *Ntn1* expressed in the ventral VZ may contribute to the CA guidance.

### Continuous expression of Ntn1 mRNAs is dispensable for CA growth

**Figure 5:**
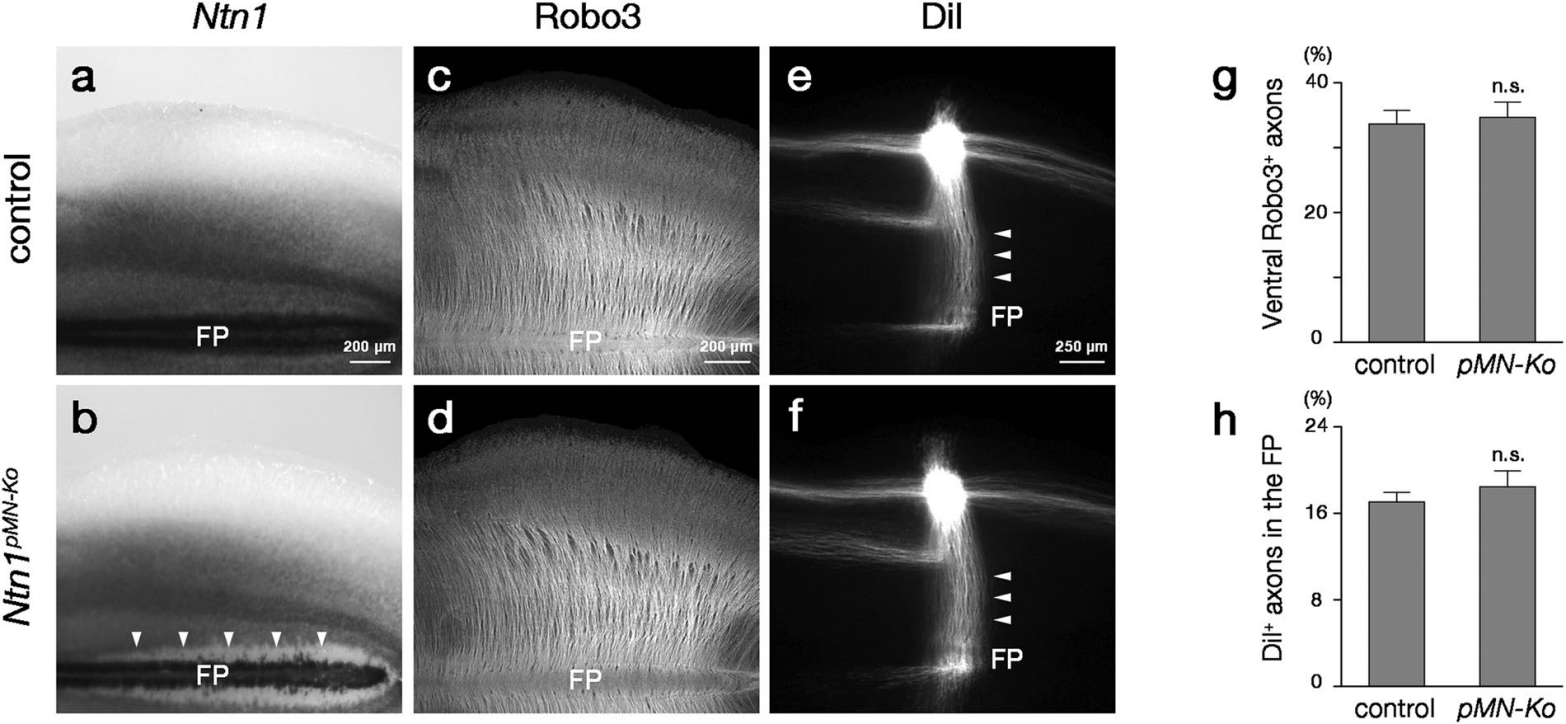
Normal CA growth in *Ntn1* ^*pMN*-*Ko*^ mice. (**a**,**b**) *Ntn1* ISH in whole-mount preparations of E12.5 control (*Olig2* ^+*/Cre*^ *;Ntn1* ^+*/flox*^) (**a**) and *Ntn1* ^*pMN*-*Ko*^ (**b**) mouse hindbrains (control, n = 6; *Ntn1* ^*pMN*-*Ko*^, n = 6). In *Ntn1* ^*pMN*-*Ko*^ mouse hindbrains, *Ntn1* hybridization signals are not detected in pMN adjacent to the FP (arrowheads). (**c**,**d**) Robo3 immunostaining in whole-mount preparations of E12.5 control (**c**) and *Ntn1* ^*pMN*-*Ko*^ (**d**) mouse hindbrains (control, n = 8; *Ntn1* ^*pMN*-*Ko*^, n = 5). In both genotypes, Robo3^+^ axons grow ventrally and reach the midline. (**e**,**f**) DiI labeling of CAs in whole-mount preparations of E12.5 control (**e**) and *Ntn1* ^*pMN*-*Ko*^ (**f**) mouse hindbrains (control, n = 8; *Ntn1* ^*pMN*-*Ko*^, n = 6). DiI-labeled CAs grow towards the midline (arrowheads) and cross it in both genotypes. (**g**,**h**) Histograms representing the fluorescence intensity of Robo3^+^ axons within the ventral one-fourth of the hindbrain normalized to that in the preparation (control, n = 8; *Ntn1* ^*pMN*-*Ko*^, n = 5; ^n.s^ *P* > 0.05, Mann–Whitney U-test; *P* = 0.724) (**g**) and the fluorescence intensity of DiI-labeled axons within the FP normalized to that at the implantation site (control, n = 8; *Ntn1* ^*pMN*-*Ko*^, n = 6; ^n.s^ *P* > 0.05, Mann–Whitney U-test; *P* = 0.573) (**h**), respectively. Error bars indicate SEM. There are no significant differences in both Robo3^+^ and DiI-labeled axon growth between genotypes. Dorsal is upwards and rostral is towards the left. Hindbrains at the rhombomere 6 to 8 level are represented. The bar in (**a**), (**c**) and (**e**) apply to (**a**,**b**), (**c**,**d**) and (**e**,**f**), respectively.

Because Ntn1 mRNA expression domain was spatially continuous along the dorsoventral axis of the VZ (Fig. 1), its continuity might be required for CAs to reach the midline. For example, Ntn1 mRNAs expressed in a dorsoventral gradient might give rise to a Ntn1 protein gradient for the CA guidance. Axon guidance by graded mRNA expressions of diffusible molecules has been proposed in other systems^32–37^. We tested this idea by deletion of *Ntn1* from somatic motor neuron progenitors (pMN) defined by *Olig2* expression^38,39^. In the *Ntn1* ^*pMN*-*Ko*^ mice (*Olig2* ^+*/Cre*^ *;Ntn1* ^*∆/flox*^ mice), Ntn1 mRNA expression was deleted from the pMN domain, creating a gap of Ntn1 mRNA expression in a region adjacent to the FP (Fig. 5b, arrowheads). However, contrary to our expectations, CAs visualized with the anti-Robo3 antibody or DiI grew normally across the gap and reached the midline (Fig. 5c–h). We confirmed that Cre-mediated recombination in the pMN domain of the *Olig2* ^*Cre*^ mice^40^ occurred around E9.5, a stage when CAs are approaching the FP (Supplementary Fig. S5). Thus, continuous expression of Ntn1 mRNAs in the VZ is dispensable for CA growth to the midline. It remains, however, to be studied how a gap of Ntn1 mRNA expression in the VZ affects Ntn1 proteins distribution.

### CA guidance to the FP is irrelevant to FP long-range chemoattraction

**Figure 6:**
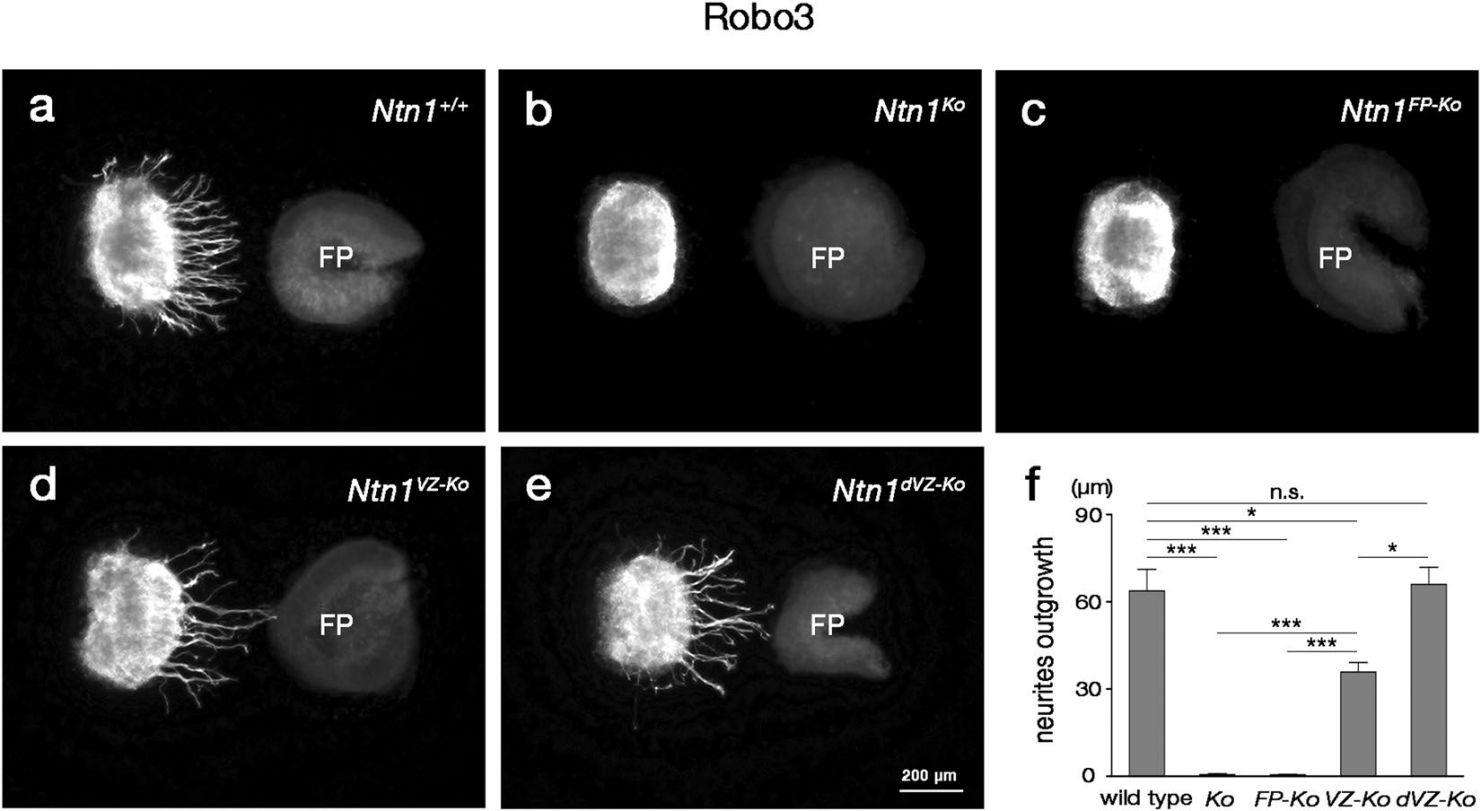
FP long-range chemoattractive activity is not correlated with CA guidance phenotypes. (**a**–**e**) Cocultures of dorsal hindbrain explants with FP explants prepared from *Ntn1* ^+/+^ (**a**), *Ntn1* ^*Ko*^ (**b**), *Ntn1* ^*FP*-*Ko*^ (**c**), *Ntn1* ^*VZ*-*Ko*^ (**d**) and *Ntn1* ^*dVZ*-*Ko*^ (**e**) mice. After culture for 28–32 h, the explants were immunostained for Robo3. FP explants from *Ntn1* ^+/+^ (**a**) and *Ntn1* ^*VZ*-*Ko*^ (**d**) and *Ntn1* ^*dVZ*-*Ko*^ mice (**e**) elicit outgrowth of Robo3^+^ neurites from the dorsal hindbrain explants, whereas few axons emanate from the dorsal hindbrain explants in coculture with FP explants from *Ntn1* ^*Ko*^ (**b**) and *Ntn1* ^*FP*-*Ko*^ (**d**) mice. (**f**) Histograms representing the Robo3^+^ neurite outgrowth from the proximal side facing the FP explant (*Ntn1* ^+/+^, n = 13; *Ntn1* ^*Ko*^, n = 10; *Ntn1* ^*FP*-*Ko*^, n = 7; *Ntn1* ^*VZ*-*Ko*^, n = 11; *Ntn1* ^*dVZ*-*Ko*^, n = 11; ^n.s.^ *P* > 0.05, *P < 0.05, ***P < 0.001, Kruskal-Wallis test followed by Steel-Dwass *post hoc* test; *P* = 5.32 × 10^−4^ for *Ntn1* ^+/+^ versus *Ntn1* ^*Ko*^, *P* = 9.83 × 10^−5^ for *Ntn1* ^+/+^ versus *Ntn1* ^*FP*-*Ko*^, *P* = 0.974 for *Ntn1* ^+/+^ versus *Ntn1* ^*dVZ*-*Ko*^). Error bars indicate SEM. FP explants from *Ntn1* ^*VZ*-*Ko*^ mice do elicit Robo3^+^ neurite outgrowth from dorsal hindbrain explants (*P* = 7.26 × 10^−4^ for *Ntn1* ^*VZ*-*Ko*^ versus *Ntn1* ^*Ko*^, *P* = 1.52 × 10^−4^ for *Ntn1* ^*VZ*-*Ko*^ versus *Ntn1* ^*FP*-*Ko*^), albeit to a lesser extent than those from *Ntn1* ^+/+^ or *Ntn1* ^*dVZ*-*Ko*^ mice (*P* = 0.0377 for *Ntn1* ^*VZ*-*Ko*^ versus *Ntn1* ^+/+^, *P* = 0.0146 for *Ntn1* ^*VZ*-*Ko*^ versus *Ntn1* ^*dVZ*-*Ko*^).

FP explants attract CAs at a distance *in vitro* by secreting diffusible axon outgrowth-promoting and tropic activities^5–10^. Ntn1 can mimic most or all of these FP activities^7,10–13^. Thus, if CA growth to the midline depends on the long-range chemoattraction by the FP, we would observe an inverse correlation between FP long-range chemoattractive activities and severities of CA guidance defects in *Ntn1* conditional mutants. We therefore examined the FP chemoattractive activities of wild-type, *Ntn1* ^*Ko*^, *Ntn1* ^*FP*-*Ko*^, *Ntn1* ^*VZ*-*Ko*^ and *Ntn1* ^*dVZ*-*Ko*^ mice using a collagen gel coculture assay^5,7,8^. Dorsal hindbrain explants from E11.5 wild-type mouse embryos were cocultured with age-matched hindbrain FP explants from *Ntn1* conditional mutants in collagen gel matrices. FP explants from wild-type, *Ntn1* ^*VZ*-*Ko*^ and *Ntn1* ^*dVZ*-*Ko*^ mice elicited Robo3^+^ neurite outgrowth from dorsal hindbrain explants, whereas those from *Ntn1* ^*Ko*^ and *Ntn1* ^*FP*-*Ko*^ mice did not (Fig. 6). Thus, the FP long-range chemoattractive activities observed *in vitro* were obviously uncorrelated with CA guidance defects in the *Ntn1* conditional mutants, arguing against the prevailing view that CA guidance to the midline is mediated by long-range diffusible chemoattractants emanating from the FP. The outgrowth-promoting activity of FP explants from *Ntn1* ^*VZ*-*Ko*^ mice appeared to be slightly lower than that of FP explants from wild-type or *Ntn1* ^*dVZ*-*Ko*^ mice (Fig. 6f). This might be because contamination of cells adjacent to the FP in the explants; these cells highly express *Ntn1* (Fig. 1d–g) but the expression can be deleted by *NestinCre*-mediated recombination (Supplementary Fig. S2).

## Discussion

**Figure 7:**
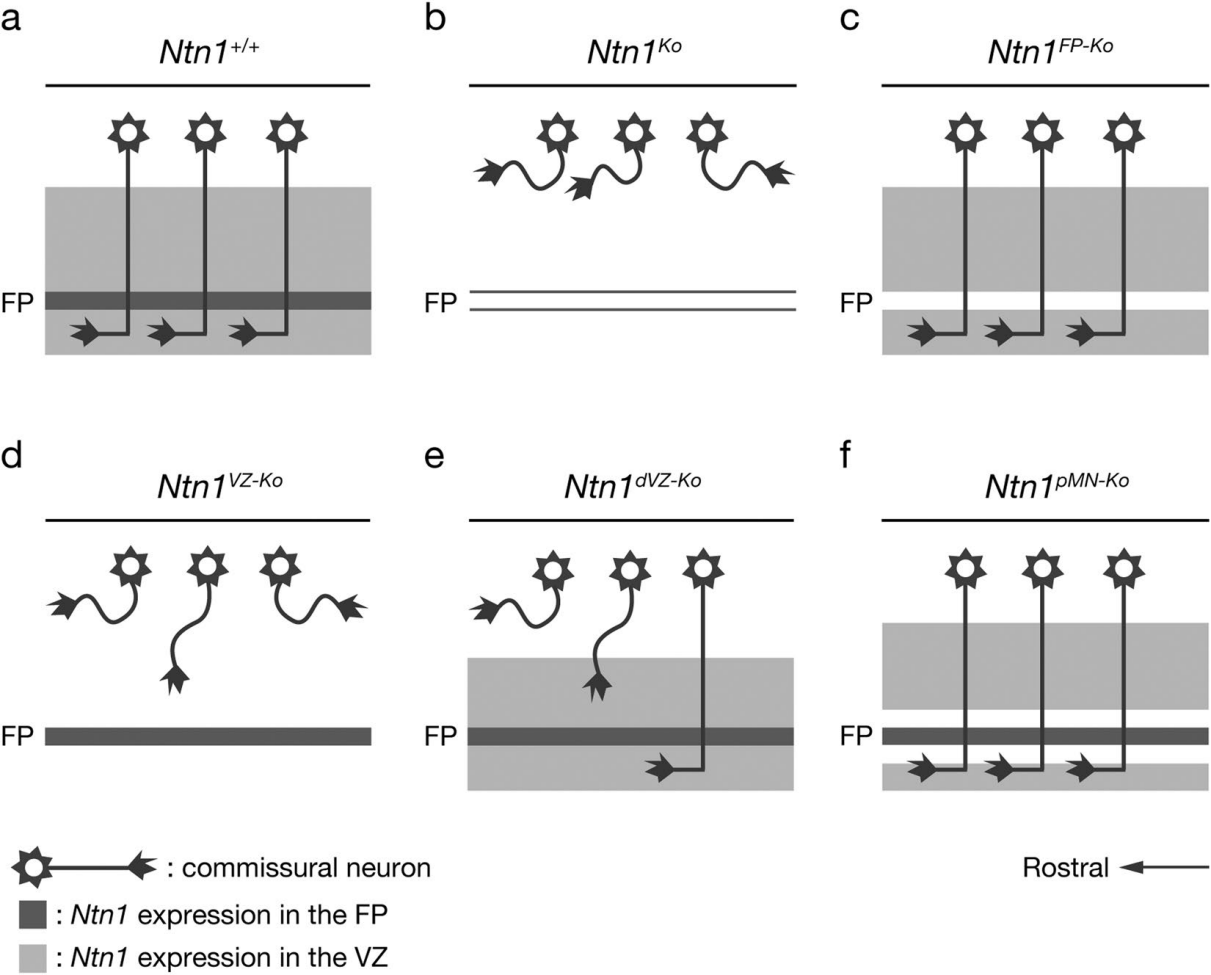
Schematics summarizing CA growth in *Ntn1* conditional mutants. *Ntn1* is expressed in the FP and the ventral two-thirds of the VZ of the hindbrain. (**a**) In wild-type mice, CAs grow straight towards the FP and crossed it. (**b**) In *Ntn1* ^*Ko*^ mice, CAs are foreshortened, spread rostrocaudally and most of them fail to invade the ventral hindbrain. (**c**) The ventrally directed growth and decussation of CAs are preserved in *Ntn1* ^*FP*-*Ko*^ mice. (**d**) *Ntn1* ^*VZ*-*Ko*^ mice exhibit CA guidance defects similar to those of *Ntn1* ^*Ko*^ mice. (**e**) The number of ventrally directed CAs is markedly reduced in *Ntn1* ^*dVZ*-*Ko*^ mice. (**f**) CAs grow normally in *Ntn1* ^*pMN*-*Ko*^ mice.

Although Ntn1 has been assumed to be an FP-derived long-range diffusible chemoattractant for CAs, here we found evidence against this notion. We showed that deletion of *Ntn1* from the VZ highly disrupts CA guidance to the midline, whereas deletion from the FP has little impact on it (Fig. 7c,d). Previous findings that CAs grow towards the midline in FP-deficient mouse embryos^18–20^ are consistent with these observations. Taken together, these findings fail to support the model that FP-derived Ntn1 acts as a long-range diffusible cue for the CA guidance, but suggest that Ntn1 derived from the VZ is crucial for CA guidance to the midline.

Our results suggest that Ntn1 acts locally on CAs to direct their ventral extension, rather than at a distance. The severe CA guidance defects caused by deletion of *Ntn1* from the dorsal VZ, where most commissural neurons differentiate (Fig. 7e), support this view. Furthermore, a previous finding that lateral neural tube fails to elicit CA outgrowth in a collagen gel coculture assay^41^ is consistent with the local action of VZ-derived Ntn1. Local Ntn actions have also been reported in other systems^42–46^. It is to be noted, however, that chemoattraction caused by a short-range diffusion of Ntn1 proteins cannot be precluded. Indeed, CAs reached the FP across a gap of *Ntn1* expression in *Ntn1* ^*pMN*-*Ko*^ mice (Fig. 7f) and CA guidance defects caused by deletion of *Ntn1* from the entire VZ were severer than those caused by deletion of *Ntn1* from the dorsal VZ (Fig. 7d,e).

VZ-derived Ntn1 at least functions as a directional cue for CAs. The anomalously rostrally deflected DiI-labeled axons observed in *Ntn1* ^*VZ*-*Ko*^ mice (Supplementary Fig. S4) and the disrupted ventral growth of Robo3^+^ axons in *Ntn1* ^*VZ*-*Ko*^ and *Ntn1* ^*dVZ*-*Ko*^ mice (Figs 3d and 4f) support this view. The findings that Ntn1 proteins exhibit a decreasing ventral-to-dorsal gradient in the neural tube^17^ and can steer CA growth *in vitro* ^7,9–11^ indicate that VZ-derived Ntn1 might regulate ventrally directed CA growth by steering their growth cones.

Dcc, Neogenin, DSCAM and Unc5s can bind Ntn1^47–49^. Of these, Dcc and Neogenin are the likely receptors for VZ-derived Ntn1 in directing CAs to the midline. A previous finding that loss of *Dcc* and *Neogenin* functions phenocopies CA guidance defects observed in the spinal cord of *Ntn1* deficient mice^50^ supports this view. Recent studies showed that *Ntn1* null mice exhibited an increased expression of Dcc and Neogenin proteins^15,16^. It would be interesting to explore how these receptors mediate CA guidance by VZ-derived Ntn1.

It seems odd that CAs grew ventrally through the mantle zone (MZ), apparently ignoring the VZ where *Ntn1* transcriptional activity was detected (Fig. 1d–g). Although Ntn1 mRNAs are expressed in the VZ, Ntn1 proteins tend to accumulate in the MZ and beneath the pial surface of the spinal cord^17^. CAs are probably guided by these Ntn1 proteins during their growth towards the midline. As the VZ is composed of radial glial cells, Ntn1 protein translated in VZ cells might be transported to the MZ through their basal processes. Indeed, Ntn1 proteins are detected in the basal processes and distal endfeet of radial glial cells^51,52^.

Although our results indicate crucial importance of VZ-derived Ntn1 for the CA guidance, the guidance defects in *Ntn1* ^*VZ*-*Ko*^ mice were less severe than those in *Ntn1* ^*Ko*^ mice (Fig. 3b,d,e). Actually, some Robo3^+^ axons still projected to the FP in *Ntn1* ^*VZ*-*Ko*^ mice (Fig. 3d). This might be attributable to the delayed onset of recombination in the *NestinCre* mice; deletion of *Ntn1* from the VZ might not be sufficiently early, so that the first cohort of CAs had already reached the FP before the deletion. Indeed, initiation of CA growth precedes *NestinCre*-mediated recombination throughout the VZ (Figs 1d,e and S2). One might argue that the residual *Ntn1* in the FP attract these axons to the midline. We, however, think it unlikely because deletion of *Ntn1* from the FP had little impact on CA growth to the midline (Fig. 7c).

Long-range chemoattraction of CAs by FP explants observed in a collagen gel coculture assay is a key piece of evidence for the FP chemoattraction model^5,7,8^. However, our observation that CA guidance was irrelevant to the FP chemoattractive activity in the collagen gel coculture assay (Fig. 6) indicates that the results measured with this assay do not necessarily reflect CA *in vivo* development. Likewise, the results obtained in a CA turning assay are inconsistent with the *in vivo* behaviors of CAs; while an FP explant taken from an *Ntn1* mutant can reorient CAs towards it when juxtaposed to the dorsal neural epithelium, CAs fail to reach the midline in the mutant^13^. Together, these findings indicate that neither of these *in vitro* assays reflects the *in vivo* behaviors of CAs. New strategies need to be developed to study *in vivo* axon guidance events^53^.

The function of FP-derived Ntn1 during CA guidance is still unclear. Our results indicate that FP-derived Ntn1 is not essential for CA guidance to the midline (Fig. 7c). A recent report has shown that Ntn1-mediated attraction from the FP guides post-crossing CAs in the rostral hindbrain^22^. However, post-crossing CA growth appears normal in the *Ntn1* ^*FP*-*Ko*^ mice (data not shown), suggesting that FP-derived Ntn1 is also dispensable for post-crossing CA guidance.

During the course of preparing this manuscript, similar findings on the role of *Ntn1* were reported by two other groups^51,52^. Dominici *et al*.^51^ also examined CA guidance in mice lacking *Ntn1* expression in the VZ or the FP and reached a conclusion similar to ours, namely that Ntn1 derived from the VZ, but not the FP, is crucial for the CA guidance. In this study, we additionally performed experiments using mice in which *Ntn1* was specifically deleted from the dorsal VZ or the pMN domain (Figs 4 and 5). Analyses of *Ntn1* ^*dVZ*-*Ko*^ mice indicated local actions of Ntn1 on CAs. We found that CAs normally develop across the *Ntn1* deleted region in *Ntn1* ^*pMN*-*Ko*^ mice (Fig. 7f), providing evidence that is difficult to explain without assuming short-distance diffusion of Ntn1 proteins. Moreover, we analyzed FP long-range chemoattractive activity of *Ntn1* conditional mutants using a collagen gel coculture assay and found that CA growth *in vivo* is irrelevant to the FP long-range chemoattration *in vitro* (Fig. 6). Varadarajan *et al*.^52^ also reported similar findings in the spinal cord. They, however, mainly used neurofilament immunostainings in their analyses, making their CA identification obscure. Both Dominici *et al*.^51^ and Varadarajan *et al*.^52^ proposed that Ntn1 promotes ventrally directed growth of CAs by haptotaxis. However, we cannot exclude the possibility that local Ntn1 diffusion contributes to the CA guidance, as discussed above.

In summary, we showed that FP-derived Ntn1 is not essential for CA guidance to the midline in the mouse hindbrain, contrary to the long-held belief that Ntn1 acts on the CA guidance as an FP-derived long-range diffusible chemoattractant. We propose a novel mechanism that VZ-derived Ntn1 acts locally on CAs to direct them to the midline.

## Methods

### Animals

All animal experiments involving animal care, surgery and sample preparation were approved by the Institutional Animal Care and Use Committees of National Institute of Genetics, Osaka University and Niigata University and conducted in accordance to Guidelines for Proper Conduct of Animal Experiments. Noon of the vaginal plug detection was designated as E0.5. Mice of either sex were used for experiments.

To generate a conditional allele of *Ntn1*, loxP sites were inserted to flank the second exon of *Ntn1* (Fig. 2a). In brief, an *Ntn1* targeting vector was constructed using C57BL/6 mouse genomic BAC clone RP23-231D12. A DNA fragment carrying a loxP site and phosphoglycerate kinase promoter-driven neomycin phosphotransferase (*pgk*-*neo*) cassette flanked by FRT sites was inserted in reverse orientation into the downstream of the exon 2. The other loxP site was inserted into the upstream of the exon 2. The floxed exon 2 with the *neo* cassette was then cloned into a vector containing 5′- and 3′- homology arms and a diphtheria toxin gene cassette. The resulting targeting vector was linearized and electroporated into a C57BL/6N ES cell line, RENKA^54^. G418-resistant clones were screened for homologous recombination by Southern blotting. The targeted clones were microinjected into host embryos as described previously^55^. Following germ-line transmission into C57BL/6N mice, the mice with the targeted allele (*Ntn1* ^*FRT*-*neo*^) were crossed with *Actb* ^*Flpe*^ mice^56^ to excise the *neo* cassette from the locus, giving rise to the conditional allele (*Ntn1* ^*flox*^). To obtain a null allele of *Ntn1* (*Ntn1* ^*∆*^), *Ntn1* ^*flox*^ mice were bred to *Foxa2* ^*iCre*^ (*Foxa2* ^*tm1*.*1(icre)Hri*^)^28^ mice, which express codon-improved Cre recombinase (iCre) in their germ cells. The other mouse strains used were; *NestinCre* (*B6*.*Cg*-*Tg(Nes*-*cre)1Kln/J*)^27^, *Ntn1* ^*LacZ*^ (*Ntn1* ^*Gt(ST629)Byg*^)^13^, *Olig2* ^*Cre*^ (*Olig2* ^*tm2(TVA*,*cre)Rth*^ */J*)^40^, *Rosa26* ^*LSLLacZ*^ (*Gt(ROSA)26Sor* ^*tm1Sor*^)^57^, *Pax3* ^*Cre*^ (*Pax3* ^*tm1(cre)Joe*^)^30^ and *Z/EG* (*Tg(CAG*-*Bgeo/GFP)21Lbe*)^29^. All mouse strains were backcrossed and maintained on an ICR background.

Mouse genotypes were determined by PCR analysis. The primer sequences for genotyping are listed in Supplementary Table S1. Genomic DNA was prepared from biopsy samples by digestion in 50 mM NaOH for 10–60 min at 95 °C followed by neutralization with 1.0 M Tris-HCl (pH 8.0). Genotyping for *Ntn1* ^*LacZ*^ and *Z/EG* alleles was also performed with X-gal histochemistry, in which tissues were soaked in the X-gal solution (see below) for 1–2 h at 37 °C.

### Hindbrain whole-mount preparation

Hindbrain whole-mount preparations were made as described previously^7^. Hindbrains were dissected in ice-cold Leibovitz’s L15 medium (Thermo Fisher Scientific) and fixed in 4% paraformaldehyde (PFA) in 0.12 M phosphate buffer (PB) (4% PFA) for 2 h to overnight at 4 °C.

### Preparation of tissue sections

Mouse tissues were collected in ice-cold phosphate-buffered saline (PBS) treated with 0.1% diethyl pyrocarbonate (DEPC) (DEPC-PBS) and immersed in 4% PFA for 2–4 h at 4 °C. After washes with DEPC-PBS, the tissues were cryoprotected in 30% sucrose in DEPC-treated 0.1 M PB overnight at 4 °C, embedded in OCT compound (Sakura Finetek) and frozen in liquid nitrogen-cooled isopentane. A series of 20- or 30-µm thick sections were cut on a cryostat (Microm HM550, Thermo Fisher Scientific or CM3050 S, Leica Microsystems) and mounted on Superfrost Plus (Fisher Scientific) or MAS-coated (Matsunami) slides.

### Culture

A collagen gel coculture assay was carried out as described previously^7,8^, with some modifications. In brief, dorsal hindbrain explants were dissected from E11.5 ICR wild-type mouse embryos. FP-containing ventral hindbrain explants (FP explants) were dissected from age-matched *Ntn1* conditional mutant mice. These tissues were prepared from the rhombomere 6–8 level, which is recognizable as an overt bulge in the caudal hindbrain. The isolated FP and dorsal hindbrain explants were embedded in collagen gel matrices at a distance of 200–400 µm as the ventromedial surface of dorsal hindbrain explants enfaced with FP explants. The cultures were grown for 28–32 h at 37 °C in Neurobasal Medium (Thermo Fisher Scientific) supplemented with N2 supplement (Thermo Fisher Scientific), 2 mM GlutaMAX I (Thermo Fisher Scientific) and penicillin/streptomycin (Thermo Fisher Scientific) and fixed in 4% PFA overnight at 4 °C.

### Antibodies

Primary antibodies used were; rabbit polyclonal anti-ß-gal antibody (1:2000, Thermo Fisher Scientific, A-11132), rabbit polyclonal anti-GFP antibody (1:1000, Thermo Fisher Scientific, A-11122) and goat polyclonal anti-Robo3 antibody (1:100–400, R&D systems, AF3076). Secondary antibodies used were; Alexa Fluor 488-conjugated donkey anti-goat IgG (1:200, Thermo Fisher Scientific, A-11055), Alexa Fluor 488-conjugated donkey anti-rabbit IgG (1:500, Thermo Fisher Scientific, A-21206), Alexa Fluor 647-conjugated donkey anti-goat IgG (1:500, Thermo Fisher Scientific, A-21447), Alexa Fluor 647-conjugated donkey anti-rabbit IgG (1:250, Thermo Fisher Scientific, A-31573) and Biotin-conjugated donkey anti-goat IgG (1:2000, Jackcon ImmunoResearch, 705–065–147).

### Immunohistochemistry

Immunohistochemistry on cryosections was carried out essentially as described previously^58,59^. In some sections, nuclei were counter-stained with propidium iodide (PI) (1:1000, Thermo Fisher Scientific) or 4′,6-diamidino-2-phenylindole (DAPI) (1:3000, Nacalai Tesque). For 3,3′-diaminobenzidine (DAB) staining, sections were reacted with avidin-biotin-horseradish peroxidase complex (1:200, ABC Elite kit, Vector Laboratories) and the peroxidase reaction was developed in 0.05% DAB (Wako), 0.003% H_2_O_2_ and 10 µM imidazole.

Whole-mount preparations were immunostained as described previously^58^, with some modifications. In brief, preparations were dehydrated and rehydrated thorough a graded series of methanol, washed in PBS containing 1.0% Triton-X 100 (1.0% PBST) and blocked with 5 or 10% horse serum (HS) in 1.0% PBST. Primary antibody incubations were performed for 2–3 d at 4 °C. After extensive washes in 1.0% PBST, the preparations were incubated with secondary antibodies for 2 d at 4 °C. The stained preparations were postfixed in 4% PFA and mounted with Mowiol (Calbiochem) containing 2.5% 1,4-diazabicyclo[2.2.2]octane (DABCO; Wako). Primary and secondary antibodies were diluted in 1.0% PBST containing 1.0% HS.

The procedures of immunostaining of explant cultures were the same as those used for whole-mount preparations, except that the methanol dehydration and rehydration steps were omitted, the Triton X-100 concentration in solutions was reduced to 0.2%, antibody incubation was performed overnight at 37 °C and counter staining was performed with DAPI.

### *In situ* hybridization

Whole-mount *in situ* hybridization (ISH) was performed as previously described^58^. cRNA probes for *Ntn1* were synthesized by *in vitro* transcription using digoxigenin labeling mix (Roche Applied Science) from a cDNA clone encoding *Ntn1* exon 2 (357–1258 bp, Genbank accession number NM_008744.2). The cDNA clone was obtained by RT-PCR from total RNA of E11.5 mouse heads and inserted into a pGEM-T easy vector (Promega).

### X-gal histochemistry

X-gal histochemistry was carried out as described previously^60^. Whole-mount preparations were fixed in 2% PFA, 0.2% glutaraldehyde and 0.02% Nonidet P-40 (NP-40) in 0.12 M PB for 1 h at 4 °C. After washes with PBS with or without 0.02% NP-40, whole-mount preparations and cryosections were treated with the X-gal solution (1 mg/ml X-gal, 5 mM K_3_Fe(CN)_6_, 5 mM K_4_Fe(CN)_6_, 2 mM MgCl_2_ and 0.02% and NP-40 in PBS) at 37 °C until color development.

### DiI labeling of CAs

DiI labeling of hindbrain CAs was performed as described previously^7,8^. A small crystal of DiI (Thermo Fisher Scientific) was implanted into the dorsal hindbrain at the rhombomere 7/8 level with a tungsten needle. Rhombomere 7/8 can be recognized as the caudal part of the bulge in the caudal hindbrain. The preparations were stored in the dark in 4% PFA for one to two weeks at room temperature. The diffusion of DiI was confirmed with the labeling of growth cones at the tips of axons.

### Image acquisition and processing

Whole-mount preparations after X-gal staining and ISH were photographed using a stereomicroscope (MZ FLIII, Leica Microsystems) equipped with a CCD camera (DP71, Olympus) at 2040 × 1536 pixel resolution. Immunofluorescence images of tissue sections and whole-mount preparations (1024 × 1024 pixels) were obtained at an optimal Z-interval with a confocal laser scanning microscope (TCS SP5, Leica Microsystems or FV-1200, Olympus). DiI-labeled axons were captured with a CCD camera (Axiocam, Zeiss) attached to an epifluorescence microscope (BX60, Olympus) at 1388 × 1040 pixel resolution. Bright-field images of tissue sections and fluorescence images of explant cultures (1920 × 1440 pixels) were acquired using a Keyence BZ-X700 epifluorescence microscope with a Z-stack module, BZ-H3XD (Keyence). Z-stack images of explant culture were captured at 14.9 µm intervals. Objective lenses used were; 2× Plan Apo; numerical aperture (NA) 0.08 (Olympus), 4× Plan Apo; NA 0.16 (Olympus) and 10× Plan Apo; NA 0.40 (Olympus) for BX-60 and FV-1200, 4× Plan Fluor; NA 0.13 (Nikon) and 10× Plan Apo; NA 0.45 (Nikon) for BZ-X700, 10× HCX Plan Apo CS; NA 0.40 (Leica Microsystems) for TCS SP5. Maximum intensity projection images were created with Leica LAS AF software (version 2.00; Leica Microsystems). Fully focused images of explant cultures, which integrate different focal planes into one image, were reconstructed using BZ-H3A software (Keyence). The brightness and contrast of images were adjusted in Adobe Photoshop CS4 or CS6.

### Quantitative analysis

To evaluate CA growth to the midline in the *Ntn1* conditional mutants, the background-subtracted fluorescence intensity of Robo3^+^ and DiI-labeled axons were measured within the ventral hindbrain and the FP, respectively, using ImageJ software (version 1.50; National Institutes of Health). The background was determined manually for each image. For quantification of Robo3^+^ axon growth, the fluorescence intensity in the ventral one-fourth of the hindbrain at the rhombomere 6–8 level was measured and normalized to that in the preparation. For quantification of DiI-labeled axons, the fluorescence intensity was measured within a 80 × 1000 µm rectangle that encompassed the center of the FP. The measured fluorescence intensity was then normalized to the fluorescence intensity at the DiI crystal implantation site to minimize variations in the size of DiI crystals.

To quantify the FP long-range chemoattractive activity for CAs in a collagen gel coculture assay, Robo3^+^ neurite outgrowth from the side facing FP explants was measured as described previously^58^. The number of pixels above the background fluorescence in neurites of the proximal side was counted using ImageJ software and then divided by the perimeter of the explant.

All statistical analyses were conducted with the aid of EZR (version 1.35; Saitama Medical Center, Jichi Medical University)^61^, which is a graphical user interface for R (The R Foundation for Statistical Computing). Mann–Whitney U-test or Kruskal-Wallis test followed by Steel or Steel-Dwass *post hoc* test was used.

### Data availability

The datasets generated during and/or analyzed during the current study are available from the corresponding author on reasonable request.

## Electronic supplementary material

Supplementary Information
