# Supplementary material for: Netrin-1 Derived from the Ventricular Zone, but not the Floor Plate, Directs Hindbrain Commissural Axons to the Ventral Midline
Source: Sci Rep. 2017 Sep 20;7:11992. doi: 10.1038/s41598-017-12269-8 (PMC5607380; doi:10.1038/s41598-017-12269-8)
Supplement: Supplementary file 1 — Supplementary Information [file 41598_2017_12269_MOESM1_ESM.pdf]

Supplementary Information

Netrin-1 Derived from the Ventricular Zone, but not the Floor Plate, Directs Hindbrain Commissural Axons to the Ventral Midline

*Kenta Yamauchi[1, 2], Maya Yamazaki[3], Manabu Abe[3], Kenji Sakimura[3], Heiko Lickert[4], Takahiko Kawasaki[1], Fujio Murakami[2], and Tatsumi Hirata[1]

[1] Division of Brain Function, National Institute of Genetics, Mishima, Shizuoka 411-8540, Japan
[2] Laboratory of Neuroscience, Graduate School of Frontier Biosciences, Osaka University, Suita, Osaka 565-0871, Japan
[3] Department of Cellular Neurobiology, Brain Research Institute, Niigata University, Niigata 951-8585, Japan
[4] Institute of Stem Cell Research and Institute of Diabetes and Regeneration Research, Helmholtz Zentrum München, D-85764 Neuherberg, Germany

Correspondence to:
Kenta Yamauchi
Division of Brain Function, National Institute of Genetics
E-mail: kntymuci@gmail.com
Phone: +81-55-981-6721, Fax: +81-55-981-6722

Supplementary Information Inventory

Supplementary Figure S1-S6
Supplementary Table S1

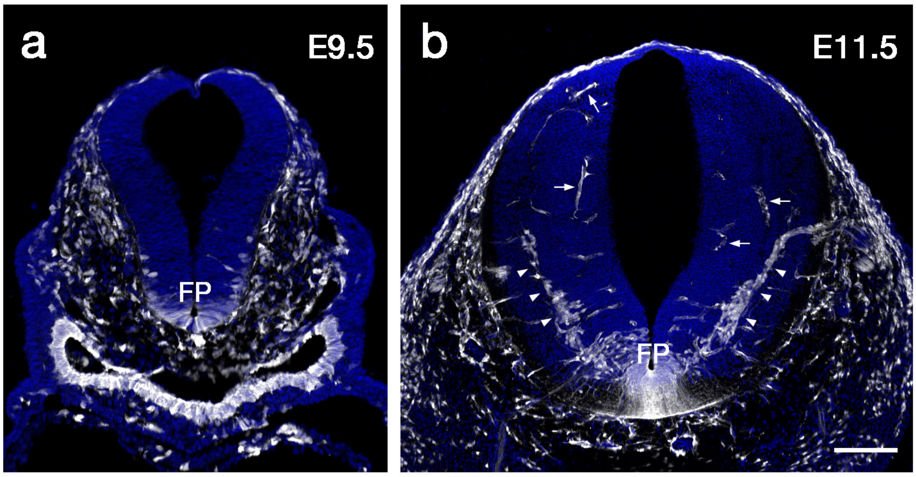

Supplementary Figure S1. EGFP reporter expression in the *Foxa2$^{+/iCre}$;ZEG* mouse hindbrain. (**a**, **b**) GFP immunostaining in E9.5 (**a**) and E11.5 (**b**) *Foxa2$^{+/iCre}$;ZEG* mouse hindbrain transverse sections (E9.5, n = 3; E11.5, n = 3). Sections are counter stained with PI (blue). EGFP reporter expression (white) is found in the FP at both E9.5 (**a**) and E11.5 (**b**). At E11.5, EGFP reporter is expressed in dorsally migrating vagal motor neurons (arrowheads), suggesting that the Cre-mediated recombination occurs in the P3 progenitor domain beyond the FP. Arrows point to blood vessels, which also express EGFP. Scale bar: (**a**) 150 µm; (**b**) 100 µm.

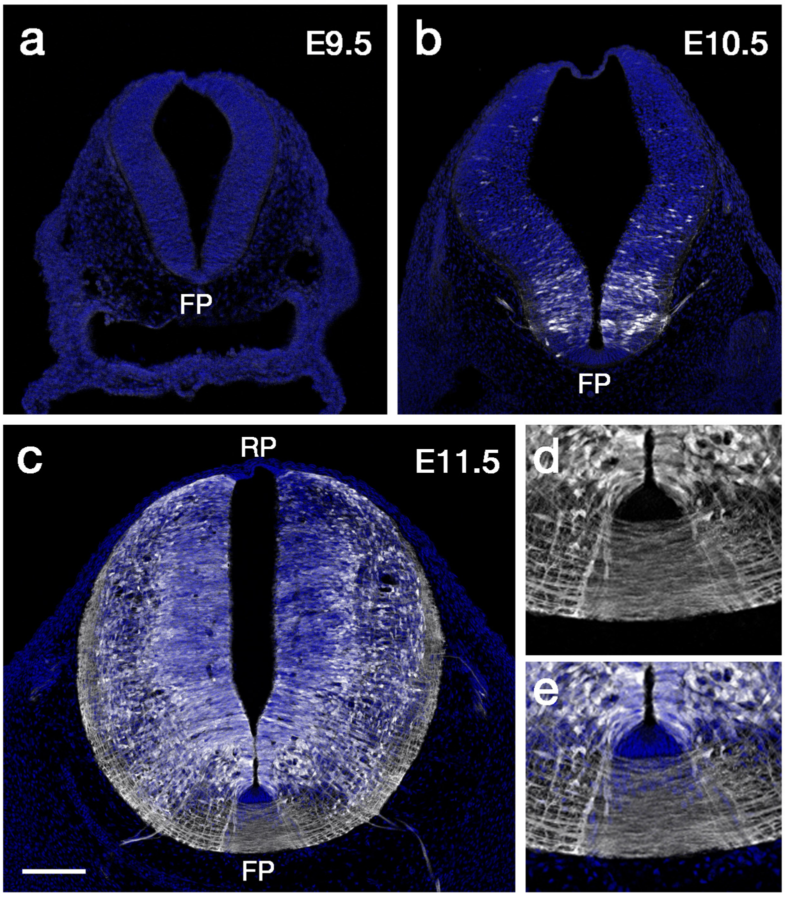

Supplementary Figure S2. EGFP reporter expression in the *NestinCre;ZEG* mouse hindbrain. (**a-c**) GFP immunostaing in E9.5 (**a**), E10.5 (**b**) and E11.5 (**c**) *NestinCre;ZEG* mouse hindbrain transverse sections (E9.5, n = 4; E10.5, n = 5; E11.5, n = 3). Nuclei are stained with PI (blue). EGFP reporter expression (white) is not detected at E9.5 (**a**) and appears by E10.5 (**b**). At E10.5, $EGFP^+$ cells are mainly located in the ventral hindbrain and scattered in the dorsal region (**b**). By E11.5, EGFP reporter expression expands throughout the neural tube except the roof plate (RP) and FP (**c**). (**d**, **e**) Enlarged views of the FP region in (**c**). (**d**) GFP immunostaining without PI staining. (**e**) A merged image of GFP immunostaining and PI staining. Note the absence of EGFP reporter expression in cell bodies of the FP. $EGFP^+$ axons crossing the midline are found at the basal region of the FP. Scale bar: (**a**) 150 μm; (**b**, **c**) 100 μm; (**d**, **e**) 200 μm.

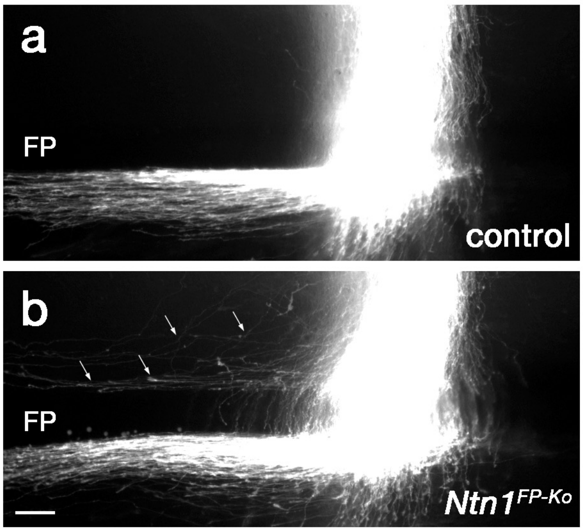

Supplementary Figure S3. Misguidance of DiI-labeled axons near the FP in *Ntn1$^{FP\text{-}Ko}$* mice. (**a**, **b**) DiI-labeled axon trajectories near the FPs of control (*Foxa2$^{+/iCre}$;Ntn1$^{+/flox}$*) (**a**) and *Ntn1$^{FP\text{-}Ko}$* mice (**b**) (control, n = 7; *Ntn1$^{FP\text{-}Ko}$*, n = 9). In control mice, DiI-labeled axons cross the FP and turn rostrally, forming a tightly fasciculated axon bundle (**a**). A limited number of axons are deflected from the bundle near the FP in *Ntn1$^{FP\text{-}Ko}$* mice (**b**). Arrows point to axons turning rostrally on the ipsilateral side. Note that the vast majority of DiI-labeled axons in the *Ntn1$^{FP\text{-}Ko}$* mice project normally. Rostral is towards the left. Scale bar: 100 μm.

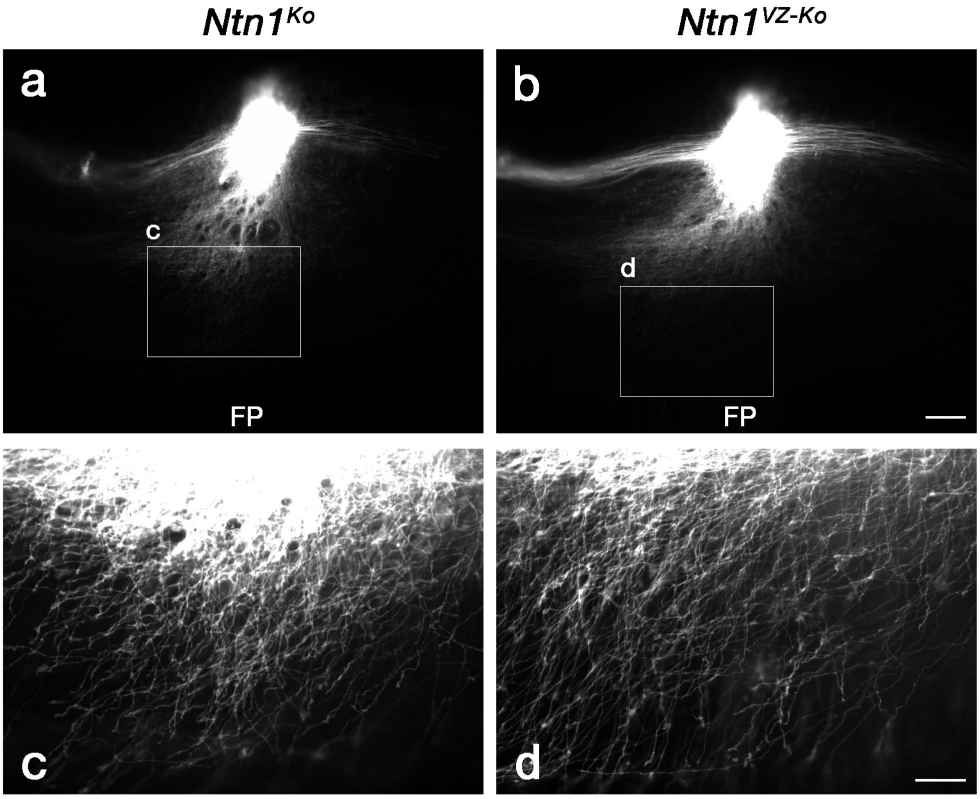

Supplementary Figure S4. Rostral deflection of DiI-labeled axons in *Ntn1*^{Ko} and *Ntn1*$^{VZ\text{-}Ko}$ mice. (**a**, **b**) DiI-labeled axon trajectories in *Ntn1*^{Ko} (**a**) and *Ntn1*$^{VZ\text{-}Ko}$ mice (**b**). (**c**) and (**d**) are higher magnification images of the areas indicated by rectangles in (**a**) and (**b**), respectively. In both genotypes, DiI-labeled axons growing in the ventral hindbrain tend to be rostrally deflected. Note that (**a**) and (**b**) are higher magnification images of Fig. 3f and h, respectively. Dorsal is upwards and rostral is towards the left. Hindbrains at the rhombomere 6-8 level are represented. Scale bars: (**a**, **b**) 100 µm; (**c**, **d**) 50 µm. The bar in (**b**) and (**d**) apply to (**a**) and (**c**), respectively.

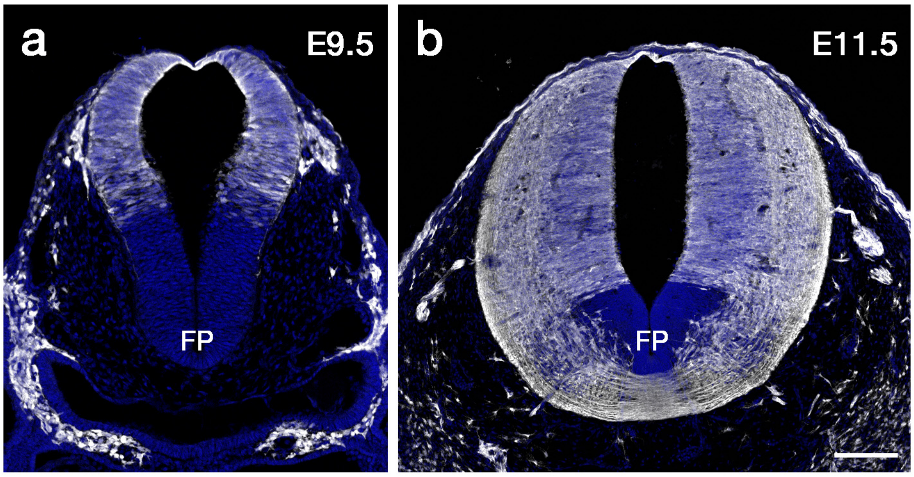

Supplementary Figure S5. EGFP reporter expression in the *Pax3$^{+/Cre}$;ZEG* mouse hindbrain. (**a**, **b**) GFP immunostaining in E9.5 (**a**) and E11.5 (**b**) *Pax3$^{+/Cre}$;ZEG* mouse hindbrain transverse sections (E9.5, n = 4; E11.5, n = 4). Sections are counter stained with PI (blue). EGFP reporter (white) is expressed in the dorsal VZ of the hindbrain at both E9.5 (**a**) and E11.5 (**b**). At E11.5, EGFP^{+} axons and cell bodies of apparent dorsal hindbrain origin invade the ventral hindbrain (**b**). Scale bar: (**a**) 150 µm; (**b**) 100 µm.

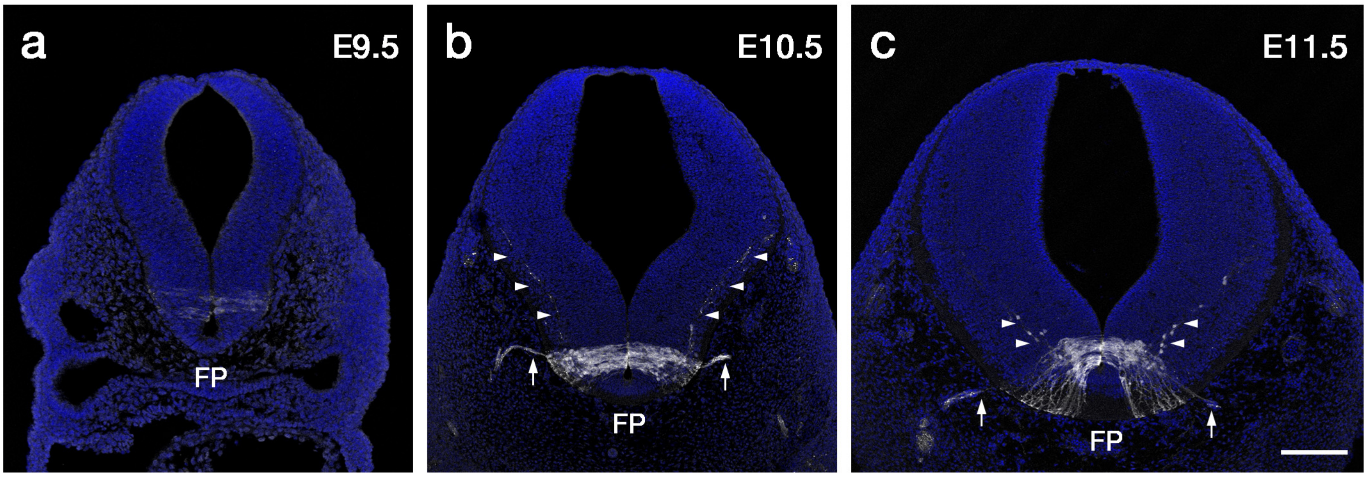

Supplementary Figure S6. ß-gal reporter expression in the *Olig2*$^{+/Cre}$*;Rosa26*$^{+/LSLLacZ}$ mouse hindbrain. (**a-c**) ß-gal immunostaining in E9.5 (**a**), E10.5 (**b**) and E11.5 (**c**) *Olig2*$^{+/Cre}$*;Rosa26*$^{+/LSLLacZ}$ mouse hindbrain transverse sections (E9.5, n = 8; E10.5, n = 3; E11.5; n = 5). Sections are counter stained with DAPI (blue). At E9.5, weak ß-gal reporter expression (white) is detected in the pMN domain adjacent to the FP in some embryos (n = 5/8, **a**), but its expression is undetectable in the other embryos. ß-gal reporter expression in the pMN domain becomes more prominent by E10.5 (**b**). Peripherally growing hypoglossal motor axons (arrows) and dorsally extending vagal motor axons (arrowheads) are also labeled with an anti-ß-gal antibody (**b**). At E11.5, ß-gal reporter expression is found in the pMN domain, hypoglossal axons (arrows) and some vagal motor neurons (arrowheads) (**c**). Scale bar: (**a**) 150 µm; (**b**, **c**) 100 µm.

Supplemental Table S1. Primer sequences for genotyping.

Alllele	*Primer name*	*Primer sequence (5'-3')*	*Product size (bp)*
Cre	*Cre F3*	*TAAAGATATCTCACGTACTGACGGTG*	*301*
	Cre R	*TCTCTGACCAGAGTCATCCTTAGC*	
Flpe	*Flpe Fw*	*CCTAAGGTCCTGGTTCGTCA*	*229*
	Flpe Rv	*TTGTTGCTTTTTGCGTCTTG*	
iCre	*iCre 250 s*	*GAGGGACTACCTCCTGTACC*	*630*
	iCre 880 as	*TGCCCAGAGTCATCCTTGGC*	
LacZ	*JL32*	*GCCGTCTGAATTTGACTGAGCGC*	*495*
	JL33	*GAAACCGACATCGCAGGCTTCTGC*	
Ntn1+	*Ntn1 Frt Fw5*	*GACATCCGCGTGGCTTTCAG*	*591*
	Ntn1 Frt Rv3	*CACTCCCTCCACCCCCTGAC*	
Ntn1FRT-neo	*Pgk pr2*	*GCTGCTAAAGCGCATGCTCC*	*369*
	Ntn1 Frt Rv3	*CACTCCCTCCACCCCCTGAC*	
Ntn1flox	*Ntn1 Frt Fw5*	*GACATCCGCGTGGCTTTCAG*	*770*
	Ntn1 Frt Rv3	*CACTCCCTCCACCCCCTGAC*	
Ntn1Δ	*Ntn1 Δ Fw2*	*GGCGGTTCAGGTTTGGAAGC*	*355*
	Ntn1 Frt Rv3	*CACTCCCTCCACCCCCTGAC*	
NestinCre	*RNE1050*	*GGCCATGAATGGAAACAGAC*	*211*
	RNE1260	*AACGCCAGTGGTTACATTCC*	
Olig2TvaIresCre	*IRES-F*	*TGGCTCTCCTCAAGCGTATT*	*~600*
	oIMR1085	*GTGAAACAGCATTGCTGTCACTT*	
Pax3Cre	*oIMR6977*	*CTGCACTCAAGGGACTCCTC*	*190*
	oIMR9074	*AGGCAAATTTTGGTGTACGG*	
Rosa26	*oIMR8545*	*AAAGTCGCTCTGAGTTGTTAT*	*Wild type: 603*
	oIMR8546	*GGAGCGGGAGAAATGGATATG*	*Mutant: 340*
	oIMR8052	*GCGAAGAGTTTGTCCTCAACC*	
